# Hyperthermia and Heat Stress as Risk Factors for Sudden Infant Death Syndrome: A Narrative Review

**DOI:** 10.3389/fped.2022.816136

**Published:** 2022-04-15

**Authors:** Véronique Bach, Jean-Pierre Libert

**Affiliations:** PeriTox, UMR_I 01, UPJV/INERIS, Jules Verne University of Picardy, Amiens, France

**Keywords:** SIDS, thermoregulation, sleep, respiration, infant, hyperthermia, thermal stress

## Abstract

**Background and Objectives:**

Heat stress and hyperthermia are common findings in sudden infant death syndrome (SIDS) victims. It has been suggested that thermal stress can increase the risk of SIDS directly *via* lethal hyperthermia or indirectly by altering autonomic functions. Major changes in sleep, thermoregulation, cardiovascular function, and the emergence of circadian functions occur at the age at which the risk of SIDS peaks—explaining the greater vulnerability at this stage of development. Here, we review the literature data on (i) heat stress and hyperthermia as direct risk factors for SIDS, and (ii) the indirect effects of thermal loads on vital physiological functions.

**Results:**

Various situations leading to thermal stress (i.e., outdoors temperatures, thermal insulation from clothing and bedding, the prone position, bed-sharing, and head covering) have been analyzed. Hyperthermia mainly results from excessive clothing and bedding insulation with regard to the ambient thermal conditions. The appropriate amount of clothing and bedding thermal insulation for homeothermia requires further research. The prone position and bed-sharing do not have major thermal impacts; the elevated risk of SIDS in these situations cannot be explained solely by thermal factors. Special attention should be given to brain overheating because of the head's major role in body heat losses, heat production, and autonomic functions. Thermal stress can alter cardiovascular and respiratory functions, which in turn can lead to life-threatening events (e.g., bradycardia, apnea with blood desaturation, and glottal closure). Unfortunately, thermal load impairs the responses to these challenges by reducing chemosensitivity, arousability, and autoresuscitation. As a result, thermal load (even when not lethal directly) can interact detrimentally with vital physiological functions.

**Conclusions:**

With the exception of excessive thermal insulation (which can lead to lethal hyperthermia), the major risk factors for SIDS appears to be associated with impairments of vital physiological functions when the infant is exposed to thermal stress.

## Introduction

Sudden infant death syndrome (SIDS) has been defined as “the sudden death of an infant under 1 year of age that remains unexplained after a thorough case investigation, including performance of a complete autopsy, examination of the death scene, and review of the clinical history” (1). A great number of factors (including the laryngeal closure reflex, sleep state disturbances, depressed arousal, apnea cerebral ischemia, and hyperthermia) have been suggested as causal in SIDS. In particular, heat stress and hyperthermia are common findings in SIDS victims. In 1984, Stanton reported that of the 34 SIDS victims studied, “19 babies were unusually hot or sweating when found dead; 14 died in an unusually warm environment; 17 had evidence of a terminal infective illness; and 24 were excessively clothed or overwrapped. In 6 of 15 babies (40%) whose rectal temperature was recorded after death, the temperature was above 37°C, the highest being 42°C” (2). Profuse sweating has been found on the scene of SIDS (3, 4), and some SIDS twins were found covered with abundant sweat (5)—a possible marker of the risk of SIDS during sleep (6). There is evidence to suggest that the risk of SIDS increases in overly hot environments, and so this aspect is an integral part of the Safe to Sleep^®^ campaign in the USA and the “Reduce the Risk”/“Back to Sleep” campaigns in other countries (7). In a multivariable regression analysis of the relationship between the overnight rectal body temperature and other variables (including putative risk factors), Tuffnell et al. (8) demonstrated that protective factors (supine position, birth weight, age, etc.) decreased the rectal temperature while risk factors (room temperature, bottle feeding, and parents who smoked) increased it. However, there is no consensus on the mechanisms that underlie the “overheating” hypothesis in SIDS.

The incidence of SIDS peaks before 4 months in preterm and term infants, respectively (9, 10). The infant is vulnerable during this period because its temperature regulation mechanisms are still developing and the rhythms of various physiological functions change after birth (11–14). Term neonates are characterized by a high body surface/body volume ratio (~0.8 cm^−1^); body heat losses to the environment are therefore greater than in older children weighing 20 kg (ratio = ~0.2 cm^−1^), for example (15). When combined with a thicker layer subcutaneous fat and an 46% increase in heat production during the first week of life (16), all the afore-mentioned anatomical and physiological characteristics augment the likelihood of excess body heat storage; the infant becomes more vulnerable to heat stress and harmful hyperthermia. Moreover, dehydration, fever, and abnormal central control of the thermoregulatory system can shorten the time to lethal hyperthermia and might thus lead to SIDS.

Infants exchange heat with the environment by radiation (between the body and the surrounding surfaces), convection (through the movement of air around the body and over the mucous membranes of the respiratory tract), conduction (*via* materials in direct contact with the skin surface) and evaporation (through transcutaneous water loss, sweating, and respiratory water losses). Body heat losses depend on the room, the radiant temperatures, the air humidity, the air flow velocity, and the clothing insulation. In France, **heat stress** has been defined as a rectal body temperature over 37.5°C, with a warning threshold of 38°C (17). When the heat load becomes too great and overcomes the effectors' thermoregulatory responses, **hyperthermia** sets in. Heat stress and hyperthermia are produced by an alteration in the body's heat balance, i.e., when body heat production and gains exceed body heat losses. This can occur following a reduction in heat dissipation from the body to the environment (mainly *via* the skin) and/or an increase in metabolic heat production. In addition to the effect of the Q_10_ temperature coefficient (a measure of a chemical reaction's temperature sensitivity, as described by van't Hoff's equation) during heat stress and hyperthermia, heat production is increased by circulating catecholamines and by the activity of the respiratory and cardiac systems. The rise in body temperature can thus be described as an accelerating system. The core body temperature increases, leading to heat stroke (over 41°C) and death (43°C has been defined as the lethal threshold) (18).

Besides the direct effects of hyperthermia, interactions between thermal stress with protective homeostatic responses might lead to potentially life-threatening events and thus SIDS during sleep. In a retrospective epidemiological study performed in the United States, Scheers-Master et al. (19) showed that heat stress was not directly and significantly related to the pathogenesis of SIDS. This finding reinforced the hypothesis whereby an elevated body temperature only acts as an additional stressor that interferes with protective homeostatic processes. Thus, it appears that heat stress alone does not cause SIDS but triggers other potentiating factors. In view of these observations, Filiano and Kinney (20) and the Task Force on Sudden Infant Death (21) suggested a triple-risk hypothesis, in which SIDS occurs in vulnerable infants exposed to environmental stressors during a critical developmental period. However, this hypothesis is subject to debate and has not been demonstrated (22).

Here, we review the literature on whether (i) thermal stress increases the risk of SIDS directly by lethal hyperthermia or indirectly *via* heat stress which induces alterations in autonomic functions, and (ii) conventional risk factors for SIDS can be interpreted in terms of the thermal load.

## Is Hyperthermia a Direct Risk Factor For SIDS?

During hyperthermia, the body core temperature is high and may lead to severe heat stress and, ultimately, death. Experiments on heated piglets showed that the hematologic, metabolic, cardiorespiratory and histological changes observed in hyperthermia were the same as those encountered in SIDS (23–25). A rapid increase in brain temperature can be associated with hemorrhagic shock and encephalopathy (26, 27). In rats, mild hyperthermia (a brain temperature of 39°C for 20 min) can produce severe ischemia in various brain structures and can induce severe neuronal necrosis (28). Hence, the hyperthermia hypothesis for SIDS is based (at least in part) on the similarities between postmortem necropsy findings and SIDS.

Hyperthermia results from the interaction of several factors, such as a high air temperature, heavy wrapping, head covering, and (sometimes) fever.

### Elevated Outdoors Temperatures and Clothing or Bedding Insulation

In a retrospective study of four US states (Georgia, Arkansas, Kansas, and Missouri) that experienced heat waves in 1980, Scheers-Masters et al. (19) reported that the daily outdoors temperature was not related to the incidence of SIDS and concluded that the association between climate and SIDS was far from consistent. However, studies in various other countries found that SIDS occurs frequently in the winter months, when the temperature outside is low (29–33). In a case-crossover analysis performed in Montreal from 1981 to 2016, Auger et al. (3) showed that after 2 months of life, SIDS was associated with an elevated outdoors temperature on the day before death and on the day of death. The discrepancies between these studies might be due to differences in infant care practices from one country to another (33). The most plausible explanation for these discrepancies relates to the fact that infants are often overwrapped and/or the parents have set an excessively high room temperature (11, 33–35), although this is also subject to debate. For air temperatures of between 15 and 25°C, Wigfield et al. (36) compared the level of clothing insulation need to maintain thermoneutrality (calculated using a mathematical model based on body heat balance equations) with the level of clothing insulation chosen by parents. The two levels were similar, and the researchers concluded that parents provided appropriate levels of thermal clothing insulation for sleep in thermal comfort, whatever the air temperature. Wailoo et al. (11) came to a similar conclusion, after reporting that in a cold British winter, the clothing insulation chosen by parents for the infants sleeping in cots was appropriate; under these conditions, the infants were able to thermoregulate and to maintain their rectal temperature within the normal range.

Values for clothing thermal insulation are found in the literature on adults (37) but are scarce in the literature on infants; the latter topic requires further research.

### Hyperthermia and the Prone Position

Prone sleeping and side sleeping positions are reportedly associated with hyperthermia (38) and SIDS (39, 40). In a study performed in New Zealand, Williams et al. (41) showed that a combination of excessive thermal insulation (>2 tog, >1.29 clo) and the prone position triggered SIDS. Similarly, Ponsonby et al. (42) reported that the risk of SIDS in prone sleepers was increased by swaddling, the use of a natural fiber mattress, recent illness, and a warm environment.

Several studies have sought to determine the thermal impact of prone sleeping on the risk of SIDS. Petersen et al. (43) compared the changes in **rectal temperature** in infants sleeping in the supine, lateral or prone position. For the prone infant, the rectal temperature did not differ significantly but tended to rise more quickly at the end of the night than for the other positions. The researchers concluded that the prone position could increase vulnerability to SIDS (43). However, the difference with the other positions was very small and would easily be compensated for by active thermoregulation. This can be explained by Tuffnell et al.'s (44) modeling of exponential body cooling; at bed-time, the **heat loss coefficient** for a prone infant was ~60% lower than those of supine and side sleepers. This is because heat loss from the head and exposed limbs is lower in the prone position than in a non-prone position. The calculated mean body temperature was the same for all the body positions. However, the non-prone sleepers reached their body temperature faster—indicating that they lost heat more rapidly than prone sleepers.

The results of many physiological studies have suggested that the prone position is associated with peripheral cutaneous vasodilation, which could increase body heat losses to the environment. Thus, Yiallourou et al. (45) have suggested that the elevated **skin temperature** found in the prone position reflects a lower level of vasomotor tone, which decreases the blood pressure and increases the heart rate. This is consistent with the lower autonomic vasoconstriction in response to a tilting test when sleeping prone (46). Longitudinal studies of infants between the ages of 2–3 weeks and 5–6 months (47) showed that **abdominal temperature** was 0.3–0.7°C higher in the prone position than in the supine position. However, the **rectal temperature** did not differ significantly when comparing the two positions. Chong et al. (48) reported that in the prone position, the **chin skin temperature** (but not the abdominal skin temperature) was higher. Skadberg and Markestad (49) observed that a distal skin temperature (measured on the left foot) during rapid-eye-movement (REM) and non-REM sleep was significantly higher in the prone position. In low-birth-weight infants (postconceptional age: 33–38 weeks), and despite the fact that the **metabolic rate** was lower in the prone position, Ammari et al. (50) observed that sleeping prone was associated with significantly higher **proximal** temperatures (+0.2°C for the forehead and flank) and **distal** temperatures (+0.4 to +0.5°C for forearm and leg) and narrowed the difference between central and peripheral temperatures (0.4°C less for forehead-to-forearm and 0.2°C less for forehead-to-environment) during both REM and non-REM sleep.

Elabbassi et al. (51) used a multisegment anthropomorphic thermal manikin (simulating a newborn with a birthweight of 1,400 g) on a plastic foam mattress to show that dry heat losses were similar in the prone and supine positions and regardless of whether the mannequin was clothed (with a diaper, a pajama, cotton swaddling, and a lightly padded sleeping bag with sleeves) or not. This is consistent with Tuffnell et al.'s (44) calculation of the same steady-state body temperature in both sleeping positions.

One can conclude that the thermal impact of the prone sleeping position is limited to higher skin temperatures and that prone sleeping does not have marked effects on internal body temperatures. Hence, it is not possible to conclude that the prone position induces hyperthermia and heatstroke.

It should be emphasized that the relationship between the prone position and thermal stress is not limited to (slight) heat stress resulting from elevated whole-body heat storage. One cannot dismiss the results of observational studies in which the prone position led to sleep modifications [more non-REM sleep, a longer sleep cycle, and higher arousal thresholds (47)], cardiorespiratory effects (higher heart and respiratory rates, lower heart and respiratory rate variability, rebreathing mechanical obstruction of the airways, and asphyxia). Although it is difficult to know whether these modifications result from the position *per se* or from the higher body temperatures in prone position, some appears to be specifically related to the body position and are independent of thermal effects (47).

### Hyperthermia and Bed-Sharing

In the review by Baddock et al. (52), bed-sharing (i.e., an infant sleeping in the same bed or on the same surface as his/her mother and sometimes his/her father) is a common practice. It is associated with positive and negative infant outcomes, which depend on the characteristics of the infant and the parents and the sleeping environment. Observations of more frequent arousals (53–55) and infant-mother interactions suggested that bed-sharing might reduce the risk of SIDS. In contrast, other studies have described bed-sharing as an unsafe sleeping environment that increases the risks of not only accidental death (e.g., suffocation) but also SIDS (56–59)—especially when the infant is sleeping with people other than the parents (59).

Several researchers have suggested that the hyperthermia induced by bed-sharing is associated with SIDS. The level of bedding thermal insulation is higher in bed-sharing and is not counterbalanced by a lower level of clothing insulation (60, 61) or by a lower room temperature. Hence, bed-sharing infants had higher levels of excessive thermal insulation than those sleeping alone (62), even after the effect of closeness to the mother's body had been taken into account. Peripheral vasodilation occurs to maintain homeothermia, with a 0.8°C increase in skin temperature [a temperature that continues to increase during the night (60) or an elevated axillary temperature during non-REM sleep (61)]. As a result, the impact on the internal body temperature is usually considered to be small [a 0.1°C increase in the rectal temperature (63)] or null (60, 64). This is in line with Young's observation [1999, cited by (53)] whereby all infants were able to regulate their body temperature. Richard (61) suggested that differences in the axillary temperature between bed-sharers and solitary sleepers during non-REM sleep only were due to homeostatic factors and not passive heating by the mother. Given that (i) the thermal impact of bed-sharing is rather small, and (ii) the interaction between bed-sharing and the thermal resistance of the infant's clothing and bedding does not significantly increase the risk of death, it appears that overdressing and hyperthermia when bed-sharing do not increase the risk of SIDS.

Interpretation of the risks associated with bed-sharing is complicated by possible additional risk factors, including the infant's age (65), cultural factors (62, 66), and maternal smoking. Many studies [but not all (59)] have concluded that the risk of SIDS is increased by bed-sharing only when the mother smokes (57, 58). According to Scragg et al. (57), 20% of all cases of SIDS in New Zealand could be explained by the combined effect of bed-sharing and maternal smoking. Similarly, Blair et al. (65) and Ruys et al. (67), respectively, reported that the risk is higher for bed-sharing infants below the age of 14 or 16 weeks.

### Hyperthermia and Head Coverings

A significant number of infants who die suddenly are found with bed covers over the head (58, 68, 69). Bacon et al. (70) pointed out that covering the infant's head with bedding increased the risk of developing hemorrhagic shock encephalopathy syndrome, which has similar pathological features to heat stroke. In a case-control study carried out in 20 European regions, Carpenter et al. (71) reported that the head was covered in 23% of deaths when sleeping. This situation was observed more frequently in prone-sleeping infants. When sleeping prone, the head remains covered because the infant cannot easily turn his/her head and/or remove the covers with his/her upper limbs and thus increase body heat losses. Moreover, an infant in the prone position can easily slip under bedding, which reduces heat losses. Sleeping bags can thus be used to prevent this risk (if appropriately used, i.e., with the right room temperature and clothing) (72).

The failure of behavioral thermoregulatory processes might be amplified by neurologic abnormalities. Korobkin and Guilleminauld (73) reported that “near-miss SIDS” infants aged under 3 months had hypotonia of the limbs and shoulder muscles, which could limit their body motility. These abnormalities disappeared with age. Blair et al. (68) systematically reviewed reports on the prevalence of head covering among SIDS victims and reported that the lack of a head covering reduced the risk of SIDS by 27.4%. The estimated risk with a head covering was five times higher than the risk in the prone position. The head is not only a major heat loss site (accounting for over 25% of the body's surface area) but is also a site of heat production (accounting for 40% of the total oxygen consumption in the brain) (74, 75). Therefore, covering the head drastically reduces heat losses by convection and radiation (which depend on vasodilation on the face) and also by evaporative skin cooling (by increasing the temperature and the humidity of the air trapped between the skin surface and the clothing). Using a mathematical model of the body heat balance that had been tested on weanling piglets covered (head and body) with infant blankets (thickness: 3 cm), Jardine and Haschke (76) showed that the time needed to raise the mean body temperature from 41°C to a lethal temperature of 43.9°C was 96 min, while removal of the blankets decreased the rectal temperature from 42 to 38°C in 82 min. After completing this experiment with a single weanling piglet, the researchers concluded that the risk of hyperthermia was zero if the entire head and a portion of the trunk's skin surface were uncovered and could lose enough heat. Similarly, Jardine (18) concluded that covered febrile infants can lose enough heat to avoid hyperthermia if a sufficient portion of the head remains uncovered. For example, the risk of hyperthermia was zero even when <30% of the head's skin surface area was exposed—as long as the blanket was not thicker than 3 cm. This finding was supported by Nelson et al.'s (38) report that in a heavily clothed infant, heat loss was particularly impaired by placing the head face down or by covering the head with bedding. Anderson et al. (77) showed that heavily covered sleeping infants can maintain normal patterns of rectal temperature as long as the head and hands are not covered.

By monitoring the rectal temperature of sleeping infants, Tuffnell et al. (8) identified low birth weight and the prone and lateral positions as major factors in SIDS, since they were associated with a higher rectal temperature. The researchers suggested that radiative heat loss from the head and the face was lower in the prone and lateral positions because contact with the insulating mattress was greater. However, it should be noted that the prone sleeping position is also associated with lower convective and evaporative heat losses. We examined this hypothesis by using a thermal manikin (nude or heavily clothed) in the prone position (face to the side) *vs*. the supine position (face straight up or face to the side) (78). When the head was not covered by a bonnet, local heat losses were similar in all positions. However, when the head was covered by a 100% acrylic bonnet (covering 85% of the head's surface area), radiative, convective and conductive heat losses from the head were greater in the face-straight-up position than in the face-to-the-side positions in which part of the head was insulated by the mattress. We calculated that the change in head position would increase the mean body temperature by 0.29°C/h for a newborn weighing 1,400 g (51). It should be noted that this increase might be much smaller for older, heavier infants (e.g., those aged 2–3 months), since the change in mean body temperature was inversely proportional to the infant's body mass. Our observation was in line with Kleemann et al.'s (4) report in which the position of the face did not play a role in SIDS: most of the infants without preterminal hyperthermia were found face down.

The results of the above-cited studies show that the association between body hyperthermia and SIDS is subject to debate. However, one cannot rule out an involvement of the brain temperature. Indeed, the brain's temperature can increase rapidly even when the core body temperature is stable (79, 80). As shown in experiments on newborn piglets (81), covering the head can also induce a lethal rise in the brain temperature. The latter temperature depends on the balance between cerebral heat production and convective heat loss *via* cooled blood flow from the vena angularis oculi. During hyperthermia, venous blood flow from the face to the sinus cavernus surrounding the posterior hypothalamus increases. This selective brain cooling mechanism (82–85) might be involved in the incidence of SIDS. When the head is entirely covered, the skin temperature of the face increases as a result of the reduced convective, radiative and evaporative heat losses. This increase might be accentuated when the infant sleeps with its face to the side because the insulating mattress impairs conductive heat loss. When the face and head skin temperatures are above the body temperature, the brain's structures (particularly the hypothalamus, which controls several vital functions) are less well cooled. Russell and Vink (86) assumed that thermoregulatory stress is a critical situation that increases the likelihood of apneic respiratory events. According to this hypothesis, REM sleep might be a critical period because animal studies have shown that the brain temperature increases during this sleep stage (87, 88). Roussel et al. (88) assumed that this rise was due to vasoconstriction. In human infants, the metabolic rate is greater during REM sleep than during non-REM sleep (89–91). All these differences might account for the higher incidence of apnea during REM sleep (92, 93).

Selective cooling of the brain *via* the vena angularis oculi might account for the data reported by Coleman-Phox et al. (94). The researchers found that in infants sleeping in the prone or lateral position at a room temperature around 21°C, the use of a fan reduced the risk of SIDS by 72%. Coleman-Phox et al. suggested that the fan reduced the build-up of carbon dioxide. However, another explanation might involve selective brain cooling; when the air temperature is below the face's skin temperature, forced ventilation around the head would increase convective and evaporative heat losses and would cool the face.

### Fever

Infection (95) and fever are frequently mentioned pathological factors in SIDS. In contrast to hyperthermia, fever increases the set-point temperature (i.e., the threshold temperature over which thermal responses are elicited); although the body's core temperature is higher than normal, it is still regulated. Many studies have reported that a mild viral infection alone is not a major risk factor for SIDS (1, 96, 97) but has a causative role when combined with heavy wrapping (clothing and bedding). In prone infants, excessive thermal insulation is associated with illness (0.93 tog, 0.60 clo). This is particularly true for SIDS victims (2.7 tog, 1.74 clo) (31). Gilbert et al. (98) reported that in heavily wrapped infants [with more than 10 tog (6.45 clo) of thermal insulation through bedding and clothing], viral infections greatly increased the risk of SIDS. Thermal stress is also magnified by the fact that the parents' response to illness (especially among less educated mothers) is often to keep their infants warm by raising the degree of thermal insulation and/or increasing the room temperature (99).

Using a mathematical model of body heat exchanges in a low-birthweight newborn wearing a bonnet and wrapped in a plastic bag, we showed that the mean body temperature increased from 40 to 43°C in 102 min (100). Metabolic heat production increased, while the mean skin temperature was kept constant (100). We also simulated acute fever during which a rise in metabolic heat production and greater peripheral vasoconstriction reduced body heat losses. The time required to reach a lethal temperature fell to 67 min. Although these results must be interpreted with a degree of caution (they relate to premature newborns, which lose heat more markedly and more quickly than older babies), they nevertheless show that lethal hyperthermia can occur rapidly in a feverish, heavily dressed infant.

Fever can thus be seen as a precipitating factor for SIDS (101). Thus, feverish infants should not be heavily clothed because thermal insulation is a key determinant of the risk of SIDS.

## Thermal Load Has Indirect Effects on Vital Physiological Functions

Along with the direct thermal effects on SIDS, prenatal and/or postnatal heat exposure can impair the autonomic nervous system. Thermal stress might thus disrupt cardiorespiratory drive and/or dampen arousal processes when a vital system is compromised.

**Prenatal heat exposure** can result in neural damage which can compromise later compensatory breathing or cardiovascular responses. Edwards et al.'s comprehensive review (102) of research on various animal species found that hyperthermia during organogenesis can have teratogenic effects. In the pregnant baboon, hyperthermia (a maternal body temperature above 41–42°C, in the absence of fever) increases fetal hypoxia, hypercapnia, acidosis, blood pressure, and heart rate (103). Maternal hyperthermia during the first trimester is associated with a greater risk of neural tube malformations and impaired brain development (104, 105). Even though data on pregnant women are rare, the few available studies also show that fetal hyperthermia (after heat exposure in a sauna or hot tub) is teratogenic (106). In feverish pregnant women, Chambers et al. (107) reported that teratogenic effects were only found for exposure with oral temperatures of 38.9°C or more and a duration >24 h in the first month of pregnancy. Although severe embryonic damage tends to lead to abortion, shorter and/or less intense heat exposures might delay the brain's development and impair its function. SIDS might thus result from *in utero* heat exposures (or other non-thermal harmful factors) *via* developmental defects in the brainstem and/or the autonomic nervous system's control of certain vital functions. Further research should seek to determine whether even subtle abnormalities can impair compensatory responses to a thermal challenge.

### Heat Stress and Cardiovascular Failure

It has been suggested that impaired cardiovascular control (i.e., failure to counter hypotension) is involved in SIDS. A number of studies have assessed the heart rate, heart rate variability (HRV), blood pressure, and blood pressure variability. Marked changes in heart rate and blood pressure control (e.g., after a head-up tilting test) can be observed when sleeping prone—especially at the age of 2–3 months, when the risk of SIDS is the greatest (45). This is consistent with the lower autonomic vasoconstriction observed in the head-up tilting test when prone (46).

Spontaneous **bradycardia** depends on thermal load in an age-dependent manner: hyperthermia enhanced the magnitude of bradycardia in 12-day-old mouse pups but not when they were younger (108).

**HRV** is often studied as a marker of the sympathetic-vagal balance; a high frequency is related to parasympathetic vagal activity, whereas a low frequency is controlled by both parasympathetic and sympathetic tones of the autonomous nervous system. Future SIDS victims are characterized by (i) lower overall HRV during REM sleep and when awake (109, 110), (ii) a greater level of sympathetic-vagal heart rate control, with a lower high-frequency power, and (iii) greater low frequency/high frequency HRV ratios (111). These features are suggestive of impaired autonomic control and might result from repeated episodes of hypoxia (111).

In a study of sleeping preterm neonates, we observed that small thermal loads (2°C below thermoneutrality) are associated with lower overall HRV (as a result of decreases in both short- and long-term variability), higher sympathetic activity, and lower parasympathetic activity—indicating that non-thermoneutral temperatures induced significant changes in autonomic nervous system control during both REM and non-REM sleep (112). Similar results have been obtained by in other studies (50, 92) and during the thermogenic phase of fever (113).

The baroreflex to **blood pressure** changes (elicited by vasoactive drugs in newborn piglets) is less sensitive during the thermogenic phase of fever (113).

### Heat Stress and Respiratory Failure

Impaired respiratory control might be involved in SIDS. Respiration is highly dependent on thermoregulation, and so thermal stress can have marked effects on the characteristics of respiratory control. Some effects are sleep-state- and age-dependent.

The **breathing rate** increases with the higher body temperature caused by fever (114), a greater environmental heat load (115) or the thermal load associated with skin-to-skin care (116). This increase in the breathing rate results from decreased respiratory drive from the thermoreceptors and thermoregulatory integrating centers in the hypothalamus. Some (but not all) studies have reported that this increase occurs during REM sleep only (117). Siren (118) has suggested that the resulting increase in the workload of the diaphragm muscles can (together with a lack of magnesium) contribute to (but not cause) the occurrence of SIDS.

An unstable breathing pattern is even observed for mild thermal stress (i.e., within the physiological temperature range). Berterottiere et al. (117) observed more frequent and longer episodes of **periodic breathing** during REM sleep only, although this pattern did not have an impact on oxygen saturation (measured using transcutaneous oximetry). It has been suggested that hyperthermia causes hyperventilation, which in turn leads to a fall in arterial CO_2_ partial pressure and then periodic breathing. Periodic breathing can be associated with clinically significant falls in cerebral oxygenation (119).

When the rectal temperature of term neonates reached 37–37.1°C, the breathing pattern was more irregular, with respiratory pauses lasting between 5 and 10 s (115). Daily et al. (120) observed that apnea was more frequent with higher skin temperatures and was only observed in conjunction with periodic breathing.

Originally, Steinschneider (121) suggested that prolonged **apnea** was part of the final pathway resulting in sudden death. The apnea theory has not, however, been proven (122). Consistently with the apnea theory, the impacts of thermal stress on apnea have been extensively studied. These studies were justified because episodes of sleep apnea are (i) longer in all sleep states in future SIDS victims, and (ii) obstructive sleep apnea is more frequent in boys (for whom the risk of SIDS is higher than for girls). Moreover, infants with obstructive apnea were more likely to sweat profusely than controls (111).

Since episodes of apnea longer than 20 s are quite rare, most of the studies concerned physiological apnea (i.e., with a shorter duration, usually from 3 s upwards). Perlstein et al. (123) observed that apnea occurred more frequently during the rising air temperature phase and assumed that this event was triggered when a thermal threshold was exceeded. In a study of healthy infants aged at least 3 weeks, we found that episodes of apnea were more frequent and longer (in REM sleep only) in a warm condition (i.e., an air temperature 2°C above the thermoneutral value) than in a cool condition (an air temperature 2°C below the thermoneutral value) (124). Bader et al.'s (125) results varied with the sleep state and the infant's age: the thermal load was associated with a greater frequency of (i) central apnea during non-REM sleep only in preterm infants and (ii) both central and obstructive apnea during REM sleep only in term infants. Similarly, in 12-week-old term neonates exposed to an air temperature of 20–30°C, Franco et al. (92) observed more frequent episodes of central apnea during REM sleep. These episodes were more often associated with blood desaturation, even though the increase in the rectal temperature was not significant. In contrast, there were no differences during non-REM sleep or for obstructive apnea.

Apnea is usually considered to be hazardous when it is accompanied by **blood bradycardia** and/or **desaturation**. Heart rate deceleration with central apnea (but not obstructive apnea) was enhanced by a higher body temperature in REM sleep only (92). As mentioned above, the thermal load increases the frequency of episodes of apnea in general and episodes with blood desaturation in particular (93). During REM sleep, warm conditions are associated with a greater frequency of episodes of apnea (especially those with blood desaturation) and more severe desaturation, relative to thermoneutral or cool conditions (92). Baddock et al. (126) reported that desaturation events were more frequent in bed-sharing infants than in those sleeping alone. Seventy percent of the desaturation events were preceded by central apnea (lasting between 5 and 10 s). In their study, the bed-sharer infants were characterized by warmer microenvironment (defined as a smaller difference between the rectal temperatures and the chin skin temperatures); the researchers calculated that a 1°C decrease in the chin-to-rectal temperature difference (i.e., a warmer environment) increased the frequency of blood oxygen desaturation by 60%. Exposure to thermal load therefore exposes the infant to repeated episodes of (mild) hypoxia, which raises the question of how the infants respond to this challenge and how these events affect the infant.

However, it is important to note that some studies failed to evidence a significant effect of thermal load on apnea or the breathing pattern, even when the skin and/or rectal temperature was higher (116, 117, 127). These apparent discrepancies might also be related to the variable chosen to quantify the thermal load. For example, Franco et al. (92) observed more statistically significant effects when considering the air temperature than when considering the rectal temperature. One of our studies might also explain these discrepancies (93). In preterm infants reaching term, we observed that episodes of apnea were more frequent in a warm condition (but only during REM sleep) and were less frequent in a cool condition (whatever the sleep state). The frequency of episodes of apnea with blood desaturation (but not that of episodes of apnea in general) was greater in the warm condition. We did not observe any significant effect on the average duration of the episodes of apnea, although the maximum duration was shorter in the cool condition. Interestingly, these comparisons of the three thermal conditions within the closed incubator differed according to whether or not apnea was considered as a function of the body's heat losses (calculated from skin, ambient and mattress temperatures, air humidity, mean radiant temperature and clothing insulation, using indirect partitional calorimetry). Our results clearly demonstrated that the frequency of episodes of apnea and the episodes' mean and maximum durations were significantly and positively correlated with body heat storage, rather than with the body temperature *per se*. This relationship was not sleep-state-dependent. These observations were consistent with Fleming et al.'s suggestion (128) that thermal effects on respiratory patterns might be linked to the detection of heat flux through the skin, since the respiratory effects usually precede skin temperature changes. Fleming et al. also suggested that the internal body temperature is not an essential component of the mechanism through which the thermal load has harmful effects on respiratory patterns. The researchers hypothesized that as a major site for heat production, heat loss, and respiratory control, the infant's head has a major role. Hence, disturbance of the thermal balance of the head alone (without a significant effect on the thermal balance of the body as a whole) might be enough to elicit impairments of breathing patterns and breathing control. Indeed, local warming of the preoptic-anterior hypothalamic area in kittens induces panting (i.e., faster breathing interspersed with periods of slower breathing) (129).

The postmortem examination of some SIDS victims evidenced chronic tissue hypoxia, which might have resulted from repeated obstruction of the airways (130). In piglets, prolonged apnea events with pathologic features similar to those observed in SIDS were elicited by the **laryngeal chemoreflex** (131). Moreover, the glottal closing force rises with the core body temperature (132). One can reasonably assume that this chemoreflex can produce asphyxia and is therefore a potential cause of SIDS if recovery processes fail (133). With regard to the impact of thermal load on this reflex, experiments in vagotomized, decerebrated piglets have demonstrated that an elevation in body temperature of between 2 and 2.5°C resulted in a longer laryngeal chemoreflex and apnea; this might contribute to SIDS (134). Haraguchi et al. (135) found that the latency and threshold of thyroarytenoid muscle activation decreased as the body temperature was increased from 34 to 41°C in anesthetized dogs (and more so in puppies than in adults). This might result from temperature-dependent changes in axonal conduction and synaptic transmission velocities. Lindgren et al. (136) pointed out that infection (associated with a 0.5°C increase in body temperature) prolonged fatal apnea through the stimulation of laryngeal chemoreflex receptors. There is now no doubt that the prolongation of this reflex by heat stress is controlled by the temperature of brain. Indeed, Van Der Velde et al. (137) showed that the rostral ventral medulla provides tonic facilitatory drive to ventilation (limiting the laryngeal reflex) and that the loss of this drive might contribute to SIDS if combined with stimuli that inhibit respiration. Xia et al. (138) reported that this thermal effect was mediated by the nucleus of the solitary tract (which contains both warm- and cold-sensitive neurons) and that the reflex was more prominent in younger animals.

The receptors in the larynx can be stimulated by liquids containing a low chloride concentration (139). When the head is covered by clothing, rebreathed water will saturate the air at body temperature and thus increase the absolute humidity of the inhaled air.

Respiratory responsiveness to experimental airway obstruction during both REM and non-REM sleep in piglets was delayed if the animal was recovering from of a respiratory tract infection. The threshold was also markedly affected, albeit during REM sleep only (140).

These effects on laryngeal sensitivity might result from the effects of hyperthermia on the cranial autonomic nerves [for a review, see (141)]. It has also been shown that the output of the respiratory neural network (as measured *via* electromyography of the diaphragm) was significantly less complex in young rats (but not in older ones) at higher body temperatures—probably as a result of impaired respiratory control (142). Nicotine exposure (another risk factor for SIDS, associated with hyperthermia) was also associated with a less complex output of the respiratory neural network (143).

It has been suggested that **breathing or rebreathing exhaled air** (i.e., the mother's breath or the infants own breath) can explain the increased incidence of SIDS in (i) bed-sharing infants [an infant lying face-to-face with the mother is exposed to air containing at least 2% CO_2_ (126)], (ii) infants with the head covered by bedding, or (iii) prone-sleeping, face-down infants (144). Using a mechanical model, Bolton et al. (145) confirmed the higher CO_2_ content near the nostrils of face-down sleeping infants. Using a geometric representation of the nostrils of an infant sleeping in the face-down position, Itzhak and Greenblatt aerodynamic study (146) demonstrated how a high-temperature environment might be a risk factor for death.

### Can Thermal Stress Impair the Response to the Cardiorespiratory Challenges That Occur Before SIDS?

It has been suggested that SIDS is due to inability to recover from prolonged apnea during sleep. Several mechanisms for recovering from sleep apnea are triggered when the chemoreceptors detect hypoxia and hypercapnia. An early-stage mechanism is arousal from sleep, whereas a late-stage mechanism involves hypoxic gasping and then autoresuscitation.

The failure of peripheral **chemosensitivity** and thus breathing control in response to prolonged apnea or to asphyxia caused by rebreathing expired air (especially in the microenvironment around the infant's mouth and nose, when the head is covered) might be involved in SIDS. An analysis of cardiorespiratory data obtained from infants who subsequently died from SIDS highlighted an alteration in the breathing response to hypoxia (147) and low chemosensitivity (148).

Oscillations in the breathing pattern (commonly observed in 1- to 3-month-old infants) can be elicited or enhanced by increasing the thermal load (128). In awake adult rats, a combination of hypothermia and severe hypoxia (7 or 11% O_2_) (but not each factor alone) inhibited respiration, whereas hyperthermia increased CO_2_ sensitivity (149). In urethane-anesthetized adult rats, responses to hypoxia or hypercapnia are also temperature-dependent: the hypoxia-hypothermia combination leads to loss of the normal response to rising CO_2_ levels during hypoventilation (150). Interestingly, when considering a warm thermal load, the response to CO_2_ differs according to whether the thermal load is due to fever or to the external environment (151). When analyzing central chemoreception in adult rats during wakefulness or non-REM sleep, Nattie and Li (152) found that the response to hypoxia was greater at 30°C (within thermoneutral zone) than at 24°C (just below the thermoneutral zone), suggesting that the mechanisms of the ventilatory response to hypoxia differ according to the thermal load. In contrast, the ventilatory responses to CO_2_ did not differ significantly at 24 vs. 30°C. In sleeping infants whose peripheral chemoreception had been tested *via* a hyperoxic test, the ventilatory response was enhanced (but not delayed) in warm or cool ambient conditions (2°C above and below the thermoneutral temperature, respectively) relative to thermoneutrality, during REM sleep but not during non-REM sleep (153). This enhancement might increase breathing instability and lead to periodic breathing or apnea (117).

SIDS is almost invariably sleep-related and so is very rare in awake infants (154). During sleep, an appropriate response to a respiratory, cardiovascular or thermal challenge may necessitate **arousal** or a change in the sleep state. Arousals are considered to be part of healthy sleep and constitute an important survival mechanism by ensuring the reversibility of sleep—especially when the infant is exposed to a life-threatening event. It has been suggested that impaired arousability is involved in SIDS (155). Therefore, several studies have investigated arousability in healthy infants or in infants with risk factors or who subsequently died from SIDS.

It has been observed that infants who subsequently died from SIDS had shorter periods of wakefulness and longer episodes of sleep than controls (156, 157). Some SIDS risk factors [the prone position, and maternal smoking (158)] are known to increase the threshold for arousal (i.e., decreased arousability). The same was observed in infants 10–15 days post-discharge from a pediatric ward after recovery from an infection (159); this finding is consistent with the increased risk of SIDS also observed at this time. In experiments on rat pups, Darnall et al. (160) demonstrated that repeated exposure to hypoxia (as might occur in some SIDS victims) decreased arousability (i.e., habituation occurred). The reverse was found for protective factors like pacifiers and breastfeeding.

Inhibition of the arousal response is accentuated by exposure to several external stressor exposures, including thermal exposure. Thermal stress can impact both spontaneous and provoked arousals. After assuming that neonates are imperfectly homeothermic organisms, Dvir et al. (161) demonstrated that ectothermic zebrafish experienced less frequent and shorter **spontaneous arousals** in hot conditions (31 or 34°C) than at an optimal water temperature (28°C). The researchers hypothesized that in neonates, a high ambient temperature reduces the neuronal noise generated by subthreshold voltage fluctuations in the wake-promoting groups of cells located in the rostral brainstem and the posterior hypothalamus (162), reducing arousability in response to a harmful situation.

During REM sleep, **arousability in response to an auditory stimulus** was greater in 3-month-old infants sleeping at 28°C than in those sleeping at 24°C. This was only seen during the third part of the night (3–6 a.m., when most SIDS deaths occur) and was not significant during non-REM sleep (163). When infants slept with their face covered, they concomitantly exhibited higher auditory arousal thresholds (in REM sleep only), a higher pericephalic ambient temperature (+2.2°C), and a higher rectal temperature (+0.24°C). The pericephalic ambient temperature was significantly and positively correlated with the arousal threshold (164, 165). However, Horne et al. (166) reported contrasting results. They observed that arousals provoked by air-jet stimulation to the nares of term infants sleeping prone were more frequent when the abdominal temperature was elevated (by 0.3–0.7°C) but not when the rectal temperature was elevated (47), or without any significant modifications of these temperatures in preterm infants. In contrast to other experts, Horne et al. hypothesized that decreased arousability when sleeping prone or after infection was independent of a thermal effect on the arousability threshold (167).

Slight hyperthermia of the brain can modify the activity of brain mediators and might therefore account for the longer sleep episodes observed in feverish patients (168). Thus, slight hyperthermia of the brain—whatever its origin—might depress arousal mechanisms.

Cardiorespiratory recordings from dying at-risk infants have shown that hypoxic **gasps** immediately precede death and that SIDS victims and infants who die of other causes differ with regard to the effectiveness and characteristics of hypoxic gasping (169).

This responsiveness might be impaired by the thermal load. In a study of a single hypoxic exposure in newborn rat pups, a higher core temperature was associated with a shorter time to the last gasp and a smaller total number of gasps (170). Similarly, hyperthermia exaggerated and extended the respiratory depression responses to hypoxia in pups exposed prenatally to cigarette smoke but not in a control (sham) group; eupneic breathing failed, gasping occurred, and recovery was attenuated (171, 172).

Sridhar et al. (169) suggested that SIDS is due to failure to **autoresuscitate** rather than failure to initiate gasping. The ability to autoresuscitate (i.e., to return to a normal heart rate and stop primary apnea) was lower at a higher core temperature when the subject was repeatedly exposed to hypoxia (170). In mice pups, a combination of hypoxia and hyperthermia prevented autoresuscitation during a single hypoxic event, whereas neither exposure alone produced similar results (173). One can conclude that thermal load (even when strictly nonlethal *per se*) affects the responses that normally prevent death during severe hypoxia and so can lead to death.

### Heat Stress and Pituitary Adenylate Cyclase-Activating Polypeptide (PACAP): A Common Mechanism?

**PACAP** is widely expressed throughout the central nervous system and is involved in many vegetative functions, including sleep (174), cardiorespiratory functions, and thermoregulation [for a review, see (175)]. There is a growing body of evidence indicates that PACAP has a role in response to challenges in infants in general and in SIDS in particular (175, 176). For an example, PACAP-deficient pups die suddenly in a manner reminiscent of SIDS (177)—probably due to defective cardiorespiratory control. Huang et al. (178) observed that PACAP levels were correlated with many SIDS risk factors (smoking, bed-sharing, infections, and seasonal temperature).

It has been demonstrated that PACAP is involved in the response to hypothermic and hyperthermic environments (175)—at least if the thermal challenge is sufficient (179). Indeed, PACAP may have an important role in the cardiorespiratory response to thermal stress. When compared with wild-type controls, PACAP-null pups exposed to severe heat stress did not exhibit the typical panting response (which increases evaporative respiratory heat losses) and showed a lower increase in the heart rate and skin temperatures (reducing heat losses from the skin and thus increasing body heat storage), somewhat greater breathing instability (as indicated by longer apnea, although not observed with other markers of breathing instability), and lower HRV. All these results argue in favor of a blunted response to heat challenges in PACAP-deficient pups, relative to controls. Barrett et al. concluded that “abnormal PACAP regulation could, therefore, contribute to neonatal disorders in which the autonomic response to heat stress is impaired, such as SIDS”.

## Conclusion and Clinical Implications

SIDS is multifactorial. Given the low incidence of SIDS, it is difficult to perform large studies of future SIDS victims—unless all infants were to undergo cardiorespiratory and hypnic recordings. In view of this difficulty, only models of SIDS can be studied. The assessment of various models might explain (at least in part) the discrepancies between some of the literature findings. Some studies looked at infants who had experienced an apparent life-threatening event, the siblings of SIDS victims, and infants with risk factors (prematurity, maternal smoking, etc.). Other studies looked at healthy infants or animals and used physical and/or mathematical models only. The cardiac, respiratory and sleep patterns recorded prior to death are not significantly abnormal, and no reliable predictors of SIDS have yet been identified.

Although many factors appear to be involved in SIDS, **thermal factors** are particularly relevant. All the studies evidently conclude that hyperthermia must be avoided and that the parents and caregivers have to pay particular attention to factors (including appropriate clothing and bedding insulation, as a function of the room temperature) that can lead to hyperthermia or heat stress and thus perturb physiological responses. In particular, little is known about the impact of the clothing thermal insulation on the development of hyperthermia and how much clothing and bedding is required to maintain the infant's thermal comfort. Appropriate guidelines on **thermal insulation** must be developed for given air temperature ranges. Only Ponsonby et al. (31) and Wigfield et al. (36) have attempted this, using mathematical models of thermal balance based on calculations of the various heat exchanges between the body and the environment. In this context, physical models like manikins (in which body heat transfers can be directly measured) avoid many uncertainties and so appear to be highly suitable.

Special attention should be paid to the risk of **brain overheating**, since large amounts of heat are lost from the head region. Reducing heat losses from the head with a blanket (18) and/or a bonnet (180) can be dangerous when the infant is heavily dressed and/or feverish.

The association between **bed-sharing** and the likelihood of heat stress and hyperthermia appears to be weak in the absence of other risk factors (such as maternal smoking, age, and cultural factors), and SIDS prevention campaigns have tended not to mention this aspect or have been inconclusive. It is nevertheless dangerous to recommend bed-sharing (due to its positive outcomes and greater mother-baby interactions) in non-smoking mothers and/or infants older than 4 months. Baddock et al. (52) have recommended reducing bedding insulation and ensuring that the infant's face and hands remain exposed, this enables heat losses and limits the thermal challenge.

Given the possible damage to the nervous system caused by heat exposure during fetal life and which might underlie SIDS, it appears necessary to protect **pregnant women** from heat. Future research projects should seek to better understand this risk (including occupational exposure) and to define danger thresholds in term of intensity and duration.

Many literature findings suggest the presence of a harmful **interaction between thermal load (even when non-lethal directly) and vital physiological functions** through the infant's autonomic nervous system. This is particularly important because at the age where the risk of SIDS peaks, the infant is undergoing major changes in sleep, thermoregulation, cardiovascular function, and the emergence of circadian functions—increasing its vulnerability. These interactions increase both the frequency and severity of autonomous challenges potentially leading to functional failure (e.g., prolonged apnea) and reduce the infant's ability to respond effectively to these vital challenges.

It should be noted that the “thermal hypothesis” does not account for all the risk factors and so requires further investigation. Factors that are known to increase the risk of SIDS but only have small effects on the thermal load should not be neglected. These include the **prone position**, which only has a small thermal impact (producing higher skin temperatures but not significantly higher internal temperatures). However, the decreased use of this sleeping position has (along with other changes induced by the various “safe to sleep” and “reduce the risk/back to sleep” campaigns) contributed to the drastic reductions in SIDS mortality worldwide (181).

Lastly, several researchers have pointed out that heat stress can act in concert with other environmental or confounding factors, such as smoking exposure. This question requires further studies and the development of mechanistic explanations with regard to the involvement of thermal and non-thermal factors in SIDS.

## Author Contributions

VB and J-PL reviewed the literature and drafted the manuscript. All authors contributed to the article and approved the submitted version.

## Conflict of Interest

The authors declare that the research was conducted in the absence of any commercial or financial relationships that could be construed as a potential conflict of interest.

## Publisher's Note

All claims expressed in this article are solely those of the authors and do not necessarily represent those of their affiliated organizations, or those of the publisher, the editors and the reviewers. Any product that may be evaluated in this article, or claim that may be made by its manufacturer, is not guaranteed or endorsed by the publisher.

## Abbreviations

PACAP: pituitary adenylate cyclase-activating polypeptide
REM: rapid eye movement sleep
SIDS: sudden infant death syndrome.
